# Comparison of Hepatocellular Carcinoma in Hispanic and Non-Hispanic Patients

**DOI:** 10.7759/cureus.14884

**Published:** 2021-05-07

**Authors:** Joseph Asemota, Olubunmi Oladunjoye, Atinuke Babalola, Ugonna Nwosu, Po-Hong S Liu, Adeolu O Oladunjoye, Nelsy Castro-Webb, Rebecca A Miksad

**Affiliations:** 1 Internal Medicine, Howard University Hospital, Washington DC, USA; 2 Hematology/Oncology, Beth Israel Deaconess Medical Center, Harvard Medical School, Boston, USA; 3 Clinical Anatomy, St. George's University School of Medicine, True Blue, GRD; 4 Internal Medicine, Tower Health-Reading Hospital, West Reading, USA; 5 Internal Medicine, UT Southwestern Medical Center, Dallas, USA; 6 Critical Care Medicine, Boston Children's Hospital, Boston, USA; 7 Epidemiology and Public Health, Boston University School of Medicine, Boston, USA; 8 Medicine, Boston University School of Medicine, Boston, USA; 9 Oncology, Flatiron Health, New York, USA

**Keywords:** hepatocellular carcinoma, hispanics, northeast united states, non-hispanic whites, hepatitis c (hcv) infection, liver cirrhosis, alcoholic liver disease, hepatitis b infection, outcome disparities, risk factors

## Abstract

Background: Hepatocellular carcinoma (HCC) is the fastest growing cancer in the United States. Studies have shown that compared to Blacks and non-Hispanic Whites, Hispanics have a higher HCC incidence and mortality rate. Most studies investigating HCC in Hispanics have been conducted utilizing data largely from the Western and Southern United States. These findings may, however, not be highly representative of Hispanics in the Northeast, given the nonhomogenous distribution and diversity of Hispanics across the United States.

Methods: Some 148 HCC patients diagnosed between 1996 and 2012 were identified from a tertiary center in the northeastern United States. Hispanic patients were randomly matched to non-Hispanic White patients by year of diagnosis. Patient characteristics, HCC risk factors, treatment, and outcome were recorded. A Kaplan-Meier (KM) plot with log-rank tests was used for survival analysis.

Results: Compared to non-Hispanic White patients (n=89), Hispanic HCC patients (n=59) were more likely to have chronic hepatitis C infection (69.5% vs. 38.2%, p < 0.01), alcoholic liver disease (37.3% vs. 21.4%, p = 0.04) and were less likely to have chronic hepatitis B infection (6.8% vs. 24.7%, p = 0.01), and private insurance (37.3% vs. 57.3%, p = 0.02). Hispanics were more likely to be diagnosed with an earlier stage disease (Barcelona Clinic Liver Cancer, BCLC stages A and B) compared to non-Hispanic patients (71.7% vs. 36.8%, p < 0.01) and were more likely to receive locoregional treatment. Although Hispanics trended towards improved overall survival, this finding did not hold when stratified by the BCLC stage.

Conclusion: Risk factors for HCC in the northeastern Hispanic population are like those found among Hispanics in other US regions. Other research suggests Hispanics are at increased risk for hepatic injury and HCC. However, HCC in this northeastern Hispanic population appears to be less aggressive (earlier stage and trend towards better overall survival) than non-Hispanics. Further research may be needed to identify potential differences by ethnic group for HCC risk factors, presentation, and outcomes.

## Introduction

Hepatocellular carcinoma (HCC) is the most common liver cancer, and it carries a poor prognosis [1]. It accounts for over 85% of all primary liver cancer and remains the third leading cause of cancer death worldwide [2]. Despite the overall decline in the incidence of cancer globally, the incidence of HCC continues to rise and it is the fastest-growing cancer in the United States [3].

There is significant geographic and ethnic variability in HCC incidence and etiologic risk factors. Globally, the highest incidence rates are seen in Asia and Africa, while much lower rates are observed in Europe and North America [2-3]. In the United States, Hispanic populations have a 2.7 times higher incidence rate and a 9% higher death rate than the non-Hispanic White population [4].

Many of the studies investigating HCC in Hispanics have focused on the Western and Southern regions of the United States. However, the country of origin for Hispanics varies by region. Hispanics from Mexico are predominant in the United States overall, but in the northeast region, Hispanic persons of Puerto Rican origin are more common [5]. In 2010, more than half of the Puerto Rican population residing in the 50 States lived in the Northeast, with more than 1.3 million Puerto Ricans living within six of the nine New England states [5].

The Surveillance, Epidemiology, and End Results (SEER) database, which is a data source for many HCC studies, includes only 2 of 18 registries (Connecticut and New Jersey) from the northeastern area [6-7]. In contrast, 14 registries from the Southern and Western areas are included [8]. Due to the geographic heterogeneity of the Hispanic population, patient characteristics for the large Puerto Rican population in New England may not be well presented in current research. This is particularly important as the prevalence of hepatitis C virus (HCV), alcohol consumption, diabetes, metabolic syndrome, and some other factors that may impact HCC diagnosis, treatment, and outcomes differ by country of origin of Hispanics [9-14].

Few HCC studies have been conducted in the Northeast where 12.6% of the US Hispanic population and 55% of Hispanics of Puerto Rico origin reside [5, 15]. Specifically, in the greater Boston area, there are 4.8 million people, 484,000 of which are Hispanics (25.9% Puerto Rican). We sought to describe and compare HCC among Hispanic and non-Hispanic patients seen at a tertiary care center in Boston, Massachusetts, which has a catchment area in the Northeast that extends to Rhode Island and New Hampshire.

## Materials and methods

Data sources

Beth Israel Deaconess Medical Center (BIDMC) is a tertiary referral center in Boston, Massachusetts. Patients were identified from the BIDMC HCC Database, a multidisciplinary, longitudinal data repository of all patients with HCC evaluated at BIDMC since 1996. Patients with a presumed HCC diagnosis were identified through the BIDMC tumor registry as well as a search of hospital electronic databases by diagnostic codes (ICD-9 155.0 and ICD-10 C22). All patients were retrospectively confirmed by pathologic and/or imaging criteria as per National Comprehensive Cancer Network® (NCCN®) HCC Guidelines 2.2014. Data recorded for each patient were: demographics, comorbidities, HCC risk factors (underlying liver disease, metabolic syndrome), tumor characteristics, as well cancer treatments and outcomes. Trained physicians abstracted structured and unstructured data from the Electronic Medical Record which were supplemented by paper chart review when necessary. Data were verified by a second physician for quality control through random secondary abstraction (about 25% of patients) as well as through missing data evaluation. Any disagreements between abstractors were arbitrated by senior physicians (A.B. and R.M.).

Inclusion criteria

Patients in the database eligible for inclusion in this study were diagnosed with HCC from 1996 to 2012. All Hispanic patients diagnosed with HCC were included. For the control group, non-Hispanic White patients were randomly chosen from the database (n=822) in a 1:1.5 ratio matched by year of diagnosis. The ratio was chosen to maximize available data.

Data abstraction methods

Individuals were identified as Hispanics using one of the following established criteria [16]: 1) A report in the electronic medical records indicating Hispanic ethnicity; 2) Spanish being listed as the preferred language in the medical records; 3) Hispanic surname as defined by the 1990 Census Spanish list. Parents’ surname (maiden name for women) was used if different from the current surname.

Metabolic syndrome was defined by the presence of at least three of the following criteria: (1) obesity (body mass index > 30 kg/m2, (2) serum triglycerides > 150 mg/dL, (3) serum high-density lipoprotein < 50 mg/dL for females and < 40 mg/dL for males, (4) history of hypertension or use of antihypertensive medications, and (5) history of diabetes or use of insulin or hypoglycemic agents. Metabolic syndrome was deemed not evaluable if there were no data documented for two or more components [17].

For alcohol, IV drugs, and tobacco, documentation of past or present use constituted exposure, regardless of quantity. If the Child-Pugh Score (CP) was not documented, it was calculated from the available recorded components. Due to the small sample size, the Barcelona Clinic Liver Cancer (BCLC) staging was trichotomized for analytical purposes. The groups were structured in order to achieve optimal similarity between patients within each group. Early stage (stage A) and intermediate stage (stage B) are herein referred to as an earlier-stage disease. Stages C and D were grouped as a late-stage disease.

Survival was defined as the time from the date of diagnosis to the date of death, last follow-up, or February 2017, whichever came first.

Statistical analysis

Data were summarized with descriptive analysis. Hispanic and non-Hispanic patients were compared using chi-square tests and Mann-Whitney U tests. Median time to death was estimated using the Kaplan-Meier (KM) life test method and compared using log-rank tests. Significance was set as a two-tailed p value < 0.05. Analyses were performed with SAS 9.4 (SAS Institute, Cary, NC).

## Results

Demographics

After matching, a total of 59 Hispanic and 89 non-Hispanic White HCC patients were included in this analysis. Baseline demographic characteristics are shown in Table 1. The median ages at diagnosis were 58.0 [range 39-84, interquartile range (IQR) 52-65 years] and 60.0 (range 24-87, IQR 52-71 years) for Hispanic and non-Hispanic Whites respectively. In our study cohort, 20.3% of Hispanic and 19.1% of non-Hispanic White HCC patients were below age 50. Hispanics (37.3%) were less likely to have private insurance when compared with the non-Hispanic White cohort (57.3%) (p = 0.02).

**Table 1:** Patient demographics. *% of the available data IVDU, intravenous drug use; TACE, trans-arterial chemoembolization; RFA, radiofrequency ablation; AFP, alpha fetoprotein; HDL, high density lipoprotein; BCLC, Barcelona Clinic Liver Cancer

Demographics	Hispanics (N=59)	Non-Hispanic Whites (N=89)	p-value
Age			
Mean age in years (SD)	59.1 (9.9)	60.3 (13.4)	0.53
Median age in years (IQR)	58.0 (52, 65)	60.0 (52, 71)	0.36
≤ 50 years	12 (20.3)	17 (19.1)	
> 50 years	47 (79.6)	72 (80.9)	
[50–64 years]	31 (52.5)	38 (42.7)	
[≥ 65 years]	16 (27.1)	34 (38.2)	
Mean weight in kilogram (SD)	28.9 (±5.5)	27.8 (±6.4)	0.01
Gender			
Male	42 (71.2)	78 (87.6)	0.01
Female	17(28.8)	11 (12.4)	
Insurance			
Public/none	37 (62.7)	38 (42.7)	0.01
Private	22 (37.3)	51 (57.3)	
Social habits			
Alcohol	43 (72.9)	55 (61.8)	0.16
Tobacco	28 (47.5)	47 (52.8)	0.52
IVDU	16 (27.1)	16 (18.0)	0.19
Metabolic syndrome components			
Hypertension	31 (52.5)	37 (42.1)	0.21
Diabetes mellitus	26 (44.1)	31 (35.2)	0.28
Obesity*	19 (38.8)	17 (25.8)	0.14
Hypertriglyceridemia*	16 (57.1)	11 (47.8)	0.51
Low HDL*	15 (50.0)	9 (37.5)	0.36
Metabolic syndrome (N=82)			
Yes	17 (46.0)	12 (26.7)	0.07
No	20 (54.1)	33 (73.3)	
Liver disease etiology			
Hepatitis B	4 (6.8)	22 (24.7)	0.01
Hepatitis C	41 (69.5)	34 (38.2)	< 0.01
Alcohol	22 (37.3)	19 (21.4)	0.04
AFP at diagnosis			
≥ 400	4 (6.9)	26 (30.6	< 0.01
< 400	54 (93.1)	59 (69.4)	
BCLC staging			
Early stage (A)	18 (34.0)	24 (27.6)	< 0.01
Intermediate stage (B)	20 (37.7)	8 (9.2)	
Late stage (C and D)	15 (28.3)	55 (63.2)	
Treatment			
TACE	18 (30.5)	8 (9.0)	< 0.01
RFA	24 (40.7)	19 (21.4)	0.01
Resection	7 (11.9)	15 (16.9)	0.48
Transplant	14 (23.7)	12 (13.5)	0.11
Systemic	15 (25.4)	27 (30.3)	0.57

Liver disease risk factors

Metabolic syndrome

Due to missing data, only 82 individuals (37 Hispanics and 45 non-Hispanic Whites) were evaluated for metabolic syndrome (Table 2). The overall prevalence of metabolic syndrome was 46.0% amongst Hispanic and 26.7% for non-Hispanic Whites (p = 0.07).

**Table 2:** Metabolic syndrome components.

Number of positive components	N (%)
None	28 (34.2%)
1	10 (12.2%)
2	15 (18.3%)
3	21 (25.6%)
4	7 (8.5%)
5	1 (1.2%)

Hepatocellular carcinoma diagnosis and treatment

Hispanics were more likely to be diagnosed with an earlier-stage HCC (71.7% vs. 36.8%, p < 0.01). They were generally more likely to receive loco-regional treatment [radiofrequency ablation (RFA), trans-arterial chemoembolization (TACE), cyberknife] compared to non-Hispanic Whites. A greater proportion of Hispanic patients than non-Hispanic Whites received RFA (40.7% vs. 21.4%, p = 0.02) and TACE (30.5% vs. 5.4%, p < 0.01). There were no significant differences between both groups in their likelihood for undergoing surgical resection (11.9% vs. 16.9%, p = 0.48), liver transplantation (23.7% vs. 13.5%, p = 0.11), or systemic therapy (25.4% vs. 30.3%, p = 0.52).

**Figure 1:**
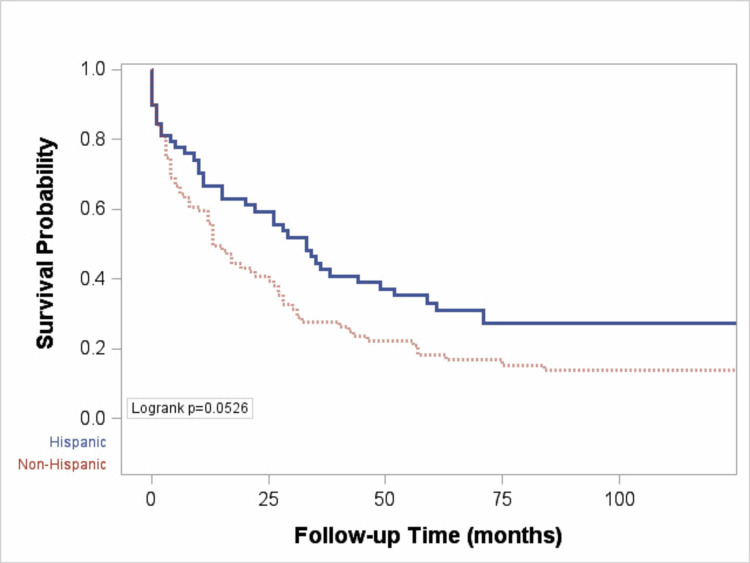
Plot comparing probability of survival of Hispanic and non-Hispanic patients with HCC. HCC, hepatocellular carcinoma

**Figure 2:**
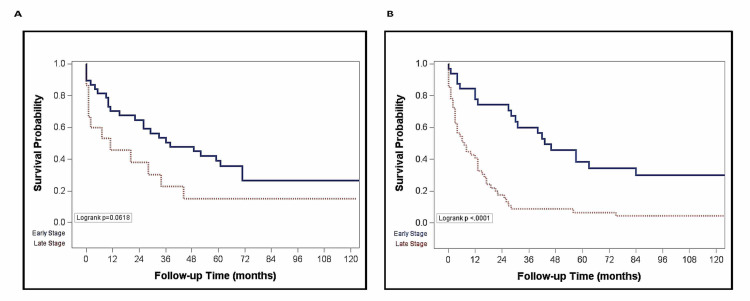
Plot comparing probability of survival by staging in Hispanic (A) and non-Hispanic (B) patients.

**Figure 3:**
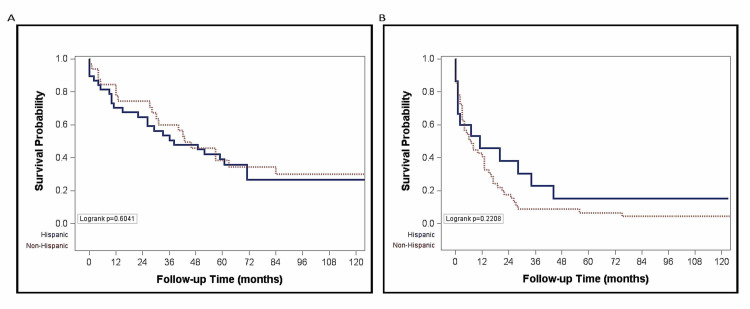
Plot of survival by ethnicity among those diagnosed at early stage (BCLC A and B) (A) and at late stage (BCLC C and D) (B). BCLC, Barcelona Clinic Liver Cancer

**Figure 4:**
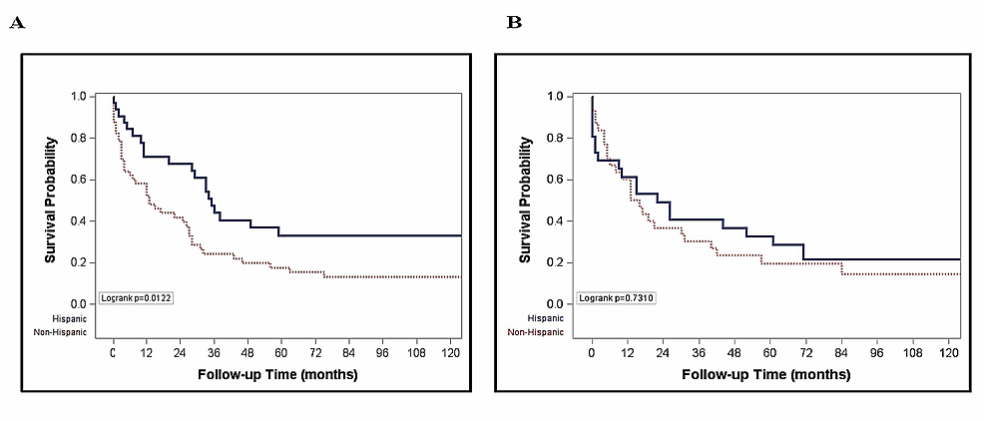
Plot of survival by ethnicity in nondiabetic (A) and diabetic (B) patients.

The overall median survival was 13 months (IQR 3-42 months) for non-Hispanic Whites and 33 months (IQR 9, 136 months) for Hispanics (p = 0.053) (Figure 1). The probability of survival at one and five years was higher (66.8% and 33.2%) for Hispanic than for non-Hispanic Whites (55.8% and 18.1%) (Figure 1). In non-Hispanic Whites, there was a longer median survival for those who presented at earlier stages (BCLC stages A and B) compared to later stages of HCC (BCLC stages C and D) (43 vs. 7 months, p < 0.01) (Figure 2B). There was however no significant difference in the median survival by ethnicity when stratified by stage at presentation (Figure 3). Among nondiabetic patients, there was a significant difference in the median survival (Hispanics, 35 months and non-Hispanics, 13 months; p = 0.01, Figure 4A).

## Discussion

Overall median survival in this cohort of northeastern Hispanics was longer compared to non-Hispanics and Hispanic Whites had a higher prevalence of HCV and alcoholic liver disease. A higher prevalence of alcoholic liver disease was observed despite no difference in the documented use of alcohol between Hispanic and non-Hispanic Whites. Together, these findings suggest a susceptibility to the hepatotoxic effects of alcohol and its metabolites among Hispanics but a more indolent HCC disease course. Although some studies have evaluated potential mechanisms, little is known about susceptibility to liver damage in Hispanics [18-19]. Our real-world evidence from a unique population in the Northeast expands knowledge about the factors influencing the incidence of and outcomes for HCC in Hispanic patients. Further evidence that suggests an increased susceptibility for hepatic injury among Hispanics is that, with alcohol consumption, Hispanics tend to have about a two-fold greater increase in aspartate aminotransferase (AST) and gamma-glutamyl transpeptidase (γ-GGT) than non-Hispanics [18]. In addition, in nonalcoholic fatty liver disease (NAFLD), hepatocyte ballooning, and formation of Mallory bodies have been shown to develop more rapidly in Hispanics [19]. A plausible explanation for the observed effect of alcohol in the Hispanic subpopulation might be the PNPLA3 gene (a genetic variant associated with increased liver fat independent of differences in body composition, diabetes or serum lipoprotein levels, and known to influence fibrogenesis related to alcoholic steatohepatitis and viral hepatitis), which occurs more commonly in Hispanics [20]. Hispanics have a higher prevalence of obesity, with increased proportions of body fat compared to total body water and this body anthropometry in Hispanics may contribute to the underlying mechanism resulting in the attainment of higher blood alcohol concentrations, ultimately leading to greater toxicity [21]. Collectively, total body fat content with the accompanying reduction in total body water, as well as PNPLA3 genetic variation might explain the greater hepatic injury susceptibility and risk of HCC among Hispanics.

Furthermore, metabolic syndrome is known to be associated with HCC [22-24] and this was seen to be more prevalent among our Hispanic cohort (46.0% vs. 26.7%). This finding is in keeping with the National Health and Nutrition Examination Survey (NHANES) data, and other studies which observed that the highest prevalence of metabolic syndrome in the United States population was within the Hispanic population [25]. Many cases of HCC without traditional risk factors are now widely attributed to metabolic syndrome [23]. A large population-based study demonstrated that pre-existing metabolic syndrome independently increased HCC risk 2.13 times [22]. Another study showed that having two components of metabolic syndrome increased the risk of HCC four-fold and each additional component increased the risk exponentially [24]. In addition, the co-existence of some of these metabolic risks with other known risk factors also has multiplicative effects. For example, co-existing obesity in chronic HBV and HCV positive patients has been associated with up to a 100-fold increased risk in HCC incidence and overall mortality [26].

The results from this study are largely in concordance with results found in other Hispanic-based HCC studies within the northeastern region and other parts of the United States. Our results suggest that compared to Non-Hispanic Whites, there is a higher prevalence of HCV infection among Hispanics (p < 0.01), which is similar to results reported by Guerrero-Preston et al. in their study looking at the risk factors for HCC among Hispanics in New York City [8]. Likewise, in a study by Wasley et al., Hispanics were shown to have a lower prevalence of HBV infection than non-Hispanic persons as was also observed in our study [27].

Finally, our analysis showed that Hispanics were less likely to have markedly elevated alpha-fetoprotein levels at the time of diagnosis of HCC. The lower levels of alpha-fetoprotein, which is often used as an early biomarker and adjunct in the diagnosis of HCC, is consistent with the finding of our Hispanic cohort being more likely to be diagnosed with earlier-stage disease. Even though previous research studies have demonstrated that Hispanics with non-alcoholic fatty liver disease have disproportionately more advanced fibrosis, cirrhosis, or HCC compared with non-Hispanics, the evidence on the comparative risk and progression of advanced liver disease and HCC in Hispanics with HCV infection remains inconclusive [28-29] and our result findings suggest a less aggressive progression of HCV-related HCC in Hispanics compared to non-Hispanic Whites. The lack of marked elevation in alpha-fetoprotein levels in Hispanics may itself be a pointer to a less active and aggressive progression of HCV-related HCC in Hispanics. More longitudinal and ethno-racial studies will, however, be needed to tease out the nuances and confirm this hypothesis. Hispanics were more likely to receive loco-regional therapy such as RFA and TACE, which are often used as palliative treatment options in patients with advanced disease in whom tumor resection is not feasible or as a bridge to liver transplantation [30-32]. Explanations for this observation might include patient-related and physician-related barriers to treatment [33]. Patient-related barriers such as personal and cultural beliefs about treatment options, as well as lower socioeconomic status and level of education could result in treatment misconceptions, which would ultimately delay early treatment initiation [33-34]. Also, physician perceptions of patient preferences, inadequate treatment work-up, immunologic mismatching of human leukocyte antigens (HLA) status, and/or delays in referral can hinder early treatment initiation [33-34].

Limitations of the study

There are several limitations to our study. First, due to the retrospective nature of our data, this study has some important limitations and thus the results should be interpreted cautiously. Second, the relatively small sample size, and the fact that our study population was drawn from a database obtained from a single center may impose limitations on the generalizability of the study findings. As a consequence of the small sample size, stratification of patients into subgroups was restricted. Third, patient ethnicities were self-identified as documented in the medical records and, as such, the veracity could not be ascertained. We also did not evaluate the effect of secondary insurance. In light of these limitations, more prospective studies are, therefore, needed to confirm the results of this study.

Despite these limitations, the study highlights the risk factors and assesses the role that these factors may play in the development of HCC among specific Hispanic populations in the northeastern United States.

## Conclusions

In conclusion, there is a disparately higher burden of hepatocellular cancer amongst Hispanics. The risk factors of HCC in the Hispanic subpopulation resident in the northeast region of the United States are largely similar to those found among Hispanics in other regions of the United States. While Hispanics show an increased susceptibility for hepatic injury and HCC, the progression of HCV-related HCC in this subpopulation appears to be less aggressive. Further research may be needed to identify potential differences by ethnic group for HCC risk factors, presentation, and outcomes.
